# Seventy-two-hour emergency department revisits among adults with chronic diseases: a Saudi Arabian study

**DOI:** 10.2147/TCRM.S168763

**Published:** 2018-08-14

**Authors:** Anwar E Ahmed, Doaa A AlBuraikan, Hend R Almazroa, Manair N Alrajhi, Bashayr I ALMuqbil, Monirah A Albaijan, Majid A Alsalamah, Hamdan AL-Jahdali

**Affiliations:** 1 King Abdullah International Medical Research Center (KAIMRC), Riyadh, Saudi Arabia, ahmeda5@vcu.edu; 2 King Saud bin Abdulaziz University for Health Sciences, National Guard Health Affairs, Riyadh, Saudi Arabia, ahmeda5@vcu.edu

**Keywords:** 72-hour ED revisits, emergency department, KAMC, Saudi Arabia

## Abstract

**Background:**

Despite the increase in adult emergency department (ED) utilization in Saudi Arabia, no studies have evaluated the 72-hour revisits. This study estimates the rate of 72-hour ED revisits and identifies its reasons and predictive factors among adults with chronic diseases.

**Patients and methods:**

A hospital-based retrospective study that included 24,206 ED discharges for adults with chronic diseases at the adult ED of King Abdulaziz Medical City, Riyadh between September 13, 2015 and July 29, 2017 was performed. We extracted data on demographic information, reasons for ED visits/revisits, health insurance coverage, weekend ED arrival, and mortality.

**Results:**

A sample of 24,206 ED discharges for 19,697 adults with at least one chronic disease was included in the analysis. The rate of 72-hour revisits in this study population was high: 3,144/24,206 (13%) had the first revisit and 319/3,144 (10.1%) had the second ED revisit within 72 hours. Diseases of the circulatory (19%) and genitourinary (15.8%) systems were the major reasons for the first ED revisit. The adjusted relative rate (aRR) of 72-hour ED revisits was higher in adults with chronic diseases and aged ≥60 years (aRR=1.360, 95% CI: 1.41–1.83; *P*=0.001), patients of female gender (aRR=1.24, 95% CI: 1.09–1.41; *P*=0.001), patients with health insurance coverage (aRR=4.23, 95% CI: 2.60–6.90; *P*=0.001), patients arriving to ED on a weekend (aRR=2.13, 95% CI: 1.03–4.41; *P*=0.041), and new patients (aRR=1.47, 95% CI: 1.25–1.73; *P*=0.001).

**Conclusion:**

The rate of 72-hour revisits is high among adults with chronic diseases. Advancing age, female gender, health insurance coverage, weekend ED arrival, and new patients are the important predictive factors of the high rate of 72-hour revisits. Continuous quality assessment and monitoring of factors related to patients are needed to reduce the frequency of early ED revisits after discharge.

## Introduction

Overcrowding the emergency department (ED) is a widespread practice in Saudi Arabia.^1^ It not only represents a large burden on the Saudi health care system, but also is a barrier for receiving timely services for urgent care,^2^,^3^ and poses great risks for cluster viral infections such as Middle East Respiratory Syndrome Coronavirus.^3^ An ED revisit within 72 hours has been used as a benchmark to improve the health care quality of services, work efficiency, and to ensure patient safety.^4^–^6^

The ED revisit within 72 hours has been widely reported in earlier studies, and its rate depends on the chronic disease status and the cultural background.^6^–^12^ The rates of 72-hour ED revisits may depend on age,^10^,^11^ gender,^10^,^13^ disease categories,^6^–^8^,^12^ medical errors,^7^,^8^ patients,^7^ and doctors.^7^ Although data regarding ED revisits within 72 hours can be used to implement changes and modifications to improve the electronic medical record database, the rate of revisits to the ED within 72 hours has not been documented among adults with chronic diseases in Saudi Arabia, particularly in our center.

This study may serve as a benchmark for the quality of urgent care in our center to reduce 72-hour revisits to the ED and improve medical care in adults with chronic diseases. The study may serve as a foundation of future studies on a national scale to assess Saudi health care system responsiveness.

This study aims to evaluate the rate, reasons, and predictive factors of the high rate of 72-hour ED revisits among adults with chronic diseases at King Abdulaziz Medical City Hospital-Riyadh (KAMC-RD), Saudi Arabia. The study aimed to evaluate the relationship between the rate of 72-hour ED revisits and age, gender, health insurance status, and day of arrival in adults with chronic diseases at KAMC-RD.

## Patients and methods

A hospital-based retrospective study was performed on the study subjects with chronic diseases who presented to the adult ED at KAMC-RD, which is the largest university hospital in Saudi Arabia.

We included all adults of age ≥18 years with at least one chronic disease who were discharged from the ED of KAMC-RD between September 13, 2015 and July 29, 2017. In 2015, KAMC-RD implemented an electronic medical record-integrated database: BESTCare. We focused on the most common chronic diseases that were set as nation health priorities for treatment and prevention, such as diabetes, heart disease, asthma, hypertension, and cancer.^14^

The following data were retrieved from the BESTCare database: age, gender, causes of ED initial visit, the date/time of ED arrival, whether a patient had a health insurance coverage, new patient (yes/no), and whether the day of ED arrival was on a weekend. Patients’ ages were divided into two groups (age group <60 and ≥60 years). A previous study forms the basis for this classification because ED revisit was found to be common in the population aged ≥60+ years.^10^ A new patient was defined as the first presentation of an individual to receive ED services.

The primary outcome of the study was the number of ED revisits within 72 hours recorded for each ED discharge (0, 1, 2, etc). The unit of analysis was the number of ED discharges. The cause of each ED revisit was collected. We used the International Statistical Classification of Diseases and Related Health Problems, Tenth Revision, Australian modification (version 2010) code, chapters I–XXII, to map and classify the cause of ED visits/revisits.

### Statistical analysis

We used IBM SPSS Statistics for Windows, version 25 (IBM Corp., Armonk, NY, USA) for data analyses. Categorical variables were presented as counts and percentages, and continuous variables as mean and SD. A univariate negative binomial regression model was used to estimate the unadjusted relative rate (RR) and to identify the factors associated with the high rate of ED revisits within 72 hours of ED discharge. A multivariate negative binomial regression model was used to estimate the adjusted relative rate (aRR) and to identify the independent factors associated with the high rate of ED revisits within 72 hours of ED discharge. The strength of the relationship was assessed by RR and aRR with 95% CI. A threshold of *P*-value ≤0.05 was used to determine the presence of statistical significance.

### Ethics approval and consent to participate

The study received ethical approval from the Ethical Review Committee at the Ministry of National Guard – Health Affairs, approval #RC17/081/R. Due to the retrospective design of the study, consent from patients to review their medical records was not required by the Ethical Review Committee at the Ministry of National Guard – Health Affairs. Data were de-identified to protect the privacy and confidentiality of patient information.

## Results

A sample of 24,206 ED discharges for 19,697 adults with at least one chronic disease was included in the analysis; 3,144/24,206 (13%) had their first revisit, of which 319 (10.1%) had a second ED revisit within 72 hours after ED discharge. Table 1 illustrates the sample and clinical characteristics. The mean age of the sample was 56.2±17.2 years, with an age range between 18 and 111.5 years.

The most common reasons for the initial ED visit among adults with chronic diseases were diseases of the circulatory system (36%), genitourinary system (31.6%), various symptoms (4.7%), respiratory system (4.2%), digestive system (4.1%), and endocrine system (3.5%).

The most common reasons for the first ED revisit within 72 hours after the initial visit were diseases of the circulatory system (19%), issues with the genitourinary system (15.8%), various symptoms (15.7%), issues with the respiratory system (12.4%), issues with the digestive system (6.5%), and issues with the endocrine system (5.7%).

**Figure 1:**
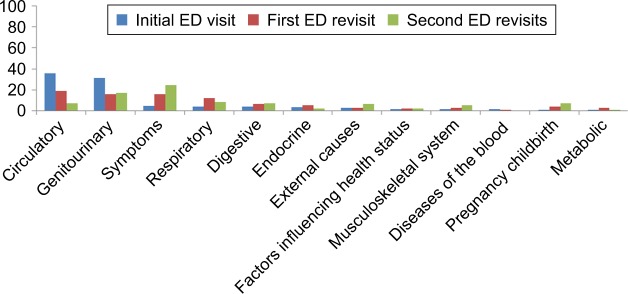
The most common reasons of ED visits and 72-hour revisits **Abbreviation:** ED, emergency department.

The most common reasons for the second ED revisit within 72 hours after the initial visit were various symptoms (24.5%), issues with the genitourinary system (17.2%), pregnancy and childbirth (8.5%), issues with the digestive system (7.2%), issues with the circulatory system (7.2%), external causes (6.9%), and issues with the musculoskeletal system (5.6%), as shown in Figure 1.

Unadjusted analysis of negative binomial (Table 2) revealed that adults with chronic diseases and aged ≥60 years (RR=1.58, 95% CI: 1.46–1.70; *P*=0.001), having health insurance coverage (RR=3.85, 95% CI: 2.74–5.38; *P*=0.001), having weekend ED arrival (RR=2.41, 95% CI: 2.15–2.69; *P*=0.001), new patients (RR=1.42, 95% CI: 1.23–1.65; *P*=0.001), and initial causes for ED visits, pregnancy and childbirth (RR=1.33, 95% CI: 1.04–1.71; *P*=0.026) increased the rate of revisits to the ED within 72 hours after ED discharge.

Adjusted analysis of negative binomial (Table 2) revealed that adults with chronic diseases and aged ≥60 years (aRR=1.60, 95% CI: 1.41–1.83; *P*=0.008), female gender (aRR=1.24, 95% CI: 1.09–1.41; *P*=0.001), having health insurance coverage (aRR=4.23, 95% CI: 2.60–6.90; *P*=0.001), having weekend ED arrival (aRR=2.13, 95% CI: 1.03–4.41; *P*=0.041), and new patients (aRR=1.47, 95% CI: 1.25–1.73; *P*=0.001) increased the rate of revisits to the ED within 72 hours after ED discharge.

## Discussion

The study evaluated the rate of frequent revisits to the ED within 72 hours by adults with chronic diseases presenting to the largest university hospital ED in Riyadh, Saudi Arabia between September 13, 2015 and July 29, 2017. Of the 24,206 ED discharges, 13% had the first ED revisit after ED discharge. This is the high rate of early ED revisit, and this trend must be explored further in a prospective study by assessing ED admission and discharge strategies.

The rate of ED revisit within 72 hours after ED discharge was considerably higher than the findings in several earlier studies that evaluated 72-hour ED revisits in the general adult populations.^7^,^8^,^10^,^15^,^16^ The rates of 72-hour return to the ED were as follows: the USA 0.5%,^16^ Taiwan 1.32%–2.38%,^8^ Singapore 2.93%,^10^ the USA 4.2%,^15^ and Taiwan 5.47%.^7^ However, the rate of ED revisits within 72 hours after ED discharge was widely reported in a recent multi-site study in the USA.^17^ Our rate falls within the site-specific range from 1.1% to 15.2% that was reported from 31 hospitals in the USA.^17^ The high rate of ED revisits within 72 hours after ED discharge in our center could be attributed to several factors related to patient, doctor, and hospital database system issues. Continuous quality assessment and monitoring of these factors may enhance the quality of ED care.

In adults with chronic diseases, several factors were found to be associated with a higher rate of ED revisits within 72 hours of discharge. For instance, the rate of ED revisits was 24% higher for females than for males. In conflict with our study, male gender was reported to be a positive predictor for ED revisits within 72 hours of discharge.^10^,^13^,^18^ It may not be possible to compare our findings with those of these studies due to methodological issues, since Ganti et al^13^ studied specific population, patients with mild traumatic brain injury. Ahmed et al^19^ studied patients with sickle cell disease. Although the bivariate results showed gender was not associated with ED revisits within 72 hours of discharge, in the multivariate results, a higher risk of ED revisits was found among patients of female gender. This discrepancy between the bivariate and multivariate analyses could be attributed to the adjustment of other factors such as elderly patient, new patient, weekend ED arrival, and health insurance coverage.

We found a higher rate of ED revisits within 72 hours of discharge by adults with chronic diseases and aged ≥60 years, consistent with the report of Chan et al^10^ and contrary to the report of Ganti et al,^13^ where an ED revisit was not associated with age. The high rate of ED revisits in adults with chronic diseases and aged ≥60 years may be due to an increased risk of developing a number of chronic diseases, which may cause unnecessary ED visits rather than seeking medical services at the primary care clinics. A study is needed in future to evaluate the access to primary care clinics and the reasons for avoiding ED visits and revisits in this population.

Unlike other studies,^13^,^17^ we found the rate of ED revisits within 72 hours of ED discharge was four times high in patients with health insurance coverage than in patients with no health insurance coverage. A number of studies have documented that there was more ED utilization in individuals with Medicaid or health insurance coverage than in uninsured or privately insured individuals.^20^–^22^

Moreover, adults with chronic diseases who visited the ED on weekends had a high rate of ED revisit within 72 hours of ED discharge. A study from Nebraska, USA, showed that the ED utilization rate was more on weekends than on workdays.^23^

The main limitation of the study is lack of several confounding factors such as patient-related reasons for ED visits and the mode of arrival to the ED. The study was a retrospective study based on a single clinical center rather than multiple clinical centers, and a prospective evaluation of 72 hours of ED revisits. The authors were not able to track ED revisits reported in another clinical center. Other confounding factors were not gathered, such as details on the mode of arrival and the number of chronic diseases. Despite these limitations, this is the first study investigating the rate of ED revisits within 72 hours of ED discharge among adults with chronic diseases in Saudi Arabia. The study utilized a sample of 24,206 ED visits and accounted for multiple revisits within 72 hours of ED discharge for each patient.

## Conclusion

The rate of 72-hour revisits among adults with chronic diseases is high in our center. Advancing age, female gender, health insurance coverage, weekend ED arrival, and new patients are predictive factors of a high rate of 72-hour revisits. Continuous quality assessment and monitoring of factors related to the patient are needed to reduce the frequency of early ED revisits after discharge. Further interventional studies are warranted to assess modifiable factors that could be beneficial to enhance ED quality care.

## Figures and Tables

**Table 1:** Characteristics and outcomes of adults with chronic diseases who visited/revisited ED within 72 hours (24,206 ED discharges)

Variable	Levels	n	%
Elderly (≥60 years)	Yes	8,646	35.8
	No	11,050	45.6
	Missing	4,510	18.6
Gender	Male	8,911	36.8
	Female	10,786	44.6
	Missing	4,509	18.6
Health insurance coverage	Yes	18,731	77.4
	No	966	4
	Missing	4,509	18.6
Weekend ED arrival	Yes	1,200	5
	No	18,496	76.4
	Missing	4,510	18.6
New patient	Yes	2,111	8.7
	No	12,541	51.8
	Missing	9,554	39.5
**Initial ED discharges (N**=**24,206)**			
Had the first revisit within 72 hours	No	21,062	87
	Yes	3,144	13
Had the second revisit within 72 hours	No	2,825	89.9
	Yes	319	10.1

**Abbreviation:** ED, emergency department.

**Table 2:** Unadjusted and adjusted relative rates of revisits to ED within 72 hours of discharge in adults with chronic diseases (24,206 ED discharges)

Factor	Bivariate analysis				Multivariate analysis			
	95% CI for RR				95% CI for aRR			
	*P*-value	RR	Lower	Upper	*P*-value	aRR	Lower	Upper
Elderly (≥60 years)	0.001^a^	1.58	1.46	1.70	0.001^a^	1.60	1.41	1.83
Female	0.461	1.03	0.95	1.11	0.001^a^	1.24	1.09	1.41
Health insurance coverage	0.001^a^	3.85	2.76	5.38	0.001^a^	4.23	2.60	6.90
Weekend ED arrival	0.001^a^	2.41	2.15	2.69	0.041^a^	2.13	1.03	4.41
New patient	0.001^a^	1.42	1.23	1.65	0.001^a^	1.47	1.25	1.73
Blood	0.103	0.81	0.63	1.04	0.382	2.47	0.33	18.67
Cause of morbidity or mortality	0.087	0.72	0.50	1.05	0.753	1.41	0.17	11.89
Kidney disease	0.052	0.68	0.46	1.00	0.362	2.60	0.33	20.43
Circulatory	0.001^a^	0.50	0.44	0.58	0.743	1.40	0.19	10.27
Digestive	0.052	0.83	0.68	1.00	0.478	2.09	0.27	15.88
Ear	0.047^a^	0.57	0.33	0.99	0.726	1.51	0.15	15.17
Endocrine	0.001^a^	0.56	0.45	0.71	0.675	1.54	0.20	11.65
External causes	0.001^a^	0.58	0.45	0.73	0.793	1.35	0.14	12.55
Eye adnexa	0.022^a^	0.62	0.41	0.93	0.939	1.09	0.12	10.14
Factors influencing health	0.001^a^	0.50	0.37	0.68	0.750	1.40	0.18	11.07
Genitourinary	0.353	0.94	0.82	1.07	0.296	2.90	0.39	21.30
Infectious	0.701	1.12	0.64	1.95	0.517	2.57	0.15	44.57
Metabolic	0.055	0.71	0.50	1.01	0.674	0.61	0.06	6.07
Musculoskeletal system	0.001^a^	0.59	0.44	0.78	0.176	0.14	0.01	2.38
Neoplasm	0.121	1.40	0.91	2.15	0.152	5.24	0.54	50.49
Nervous	0.246	0.74	0.45	1.23	0.333	3.39	0.29	40.03
Pregnancy childbirth	0.026^a^	1.33	1.04	1.71	0.110	5.90	0.67	51.96
Respiratory	0.013^a^	0.78	0.64	0.95	0.754	0.70	0.08	6.48
Skin subcutaneous	0.459	0.84	0.53	1.34	1.000	1.00	0.06	16.74
Others	0.017^a^	0.60	0.40	0.91	0.285	3.14	0.39	25.51

**Note:**

a Significant at *α*=0.05.

**Abbreviations:** aRR, adjusted relative risk; ED, emergency department; GU, genitourinary; MSK, musculoskeletal system; RR, relative risk.
